# Correlation of limited-early-response status with 12-month CST, BVA, and machine learning-quantified retinal fluid in diabetic macular oedema in routine clinical practice

**DOI:** 10.1038/s41433-024-03172-4

**Published:** 2024-06-18

**Authors:** Resya C. Sastry, Scott W. Perkins, Aneesha Kalur, Rishi P. Singh

**Affiliations:** 1 Center for Ophthalmic Bioinformatics, Cole Eye Institute, Cleveland Clinic, Cleveland, OH USA; 2 Cleveland Clinic Lerner College of Medicine, Cleveland, OH USA

**Keywords:** Eye diseases, Retinal diseases

## Abstract

**Background/Objectives:**

Anti-VEGF treatment response in DMO has been measured by changes in the central subfield thickness (CST) and best visual acuity (BVA) outcomes at 3 months after initial treatment, termed early or limited early response (ER/LER). This study correlates LER with 12-month BVA, CST, and retinal fluid volumes quantified by a machine learning algorithm on optical coherence tomography (OCT).

**Subjects/Methods:**

The study included treatment naïve DMO patients ≥ 18 years with OCT scans at baseline (M0), M3, M6, and M12. The 220 patients were categorized as limited early responders (LER) if they had ≤ 10% CST reduction and/or < 5 ETDRS letter gain at M3. BVA, CST, and subretinal (SRF), intraretinal (IRF), and total retinal (TRF) fluid volumes quantified by a machine learning algorithm were compared between groups and across time.

**Results:**

At M12, the anatomic LER (aLER), defined solely by CST, had significantly worse BVA and CST versus the anatomic ER (aER) group (*p* < 0.001). Retinal fluid M12 outcomes did not significantly vary between all LER and ER groups. No significant BVA, CST, TRF, and IRF variance across time for LER was found (*p* > 0.1).

**Conclusions:**

BVA and CST M12 outcomes vary by aLER/aER status indicating that CST may be a strong predictor of treatment outcomes, while retinal fluid volumes were not predicted by LER status.

## Introduction

Diabetic Macular Oedema (DMO) is a leading cause of vision loss among patients with diabetes mellitus (DM). DMO is characterized by accumulation of fluid within the macula, often due to the breakdown of the blood-retinal barrier (BRB) [1]. Associative risk factors for DMO include high haemoglobin A_1C_ levels and those with longer duration of DM [2]. DMO commonly develops in patients with diabetic retinopathy (DR) due to vascular permeability [3, 4]. Increased serum glucose leads to BRB disruption, increasing ischemic drive and upregulating vascular endothelial growth factors (VEGF) and cytokines, which leaks plasma solutes into interstitial spaces causing edema [5–8]. The primary treatment for DMO are anti-VEGF injections, such as ranibizumab, bevacizumab, and aflibercept, as they demonstrate better best visual acuity (BVA) outcomes relative to laser treatment [9, 10].

Anti-VEGF early treatment response is assessed anatomically by changes in the central subfield thickness (CST) and visually by best corrected visual acuity (BCVA) outcomes at 3 months after initial monthly injection. Those with ≤ 10% CST reduction and/or a BCVA gain of < 5 ETDRS letters 3 months after initial treatment are classified as limited-early responders, while those who do not fit the criteria are early responders [11]. Piermaci et al.’s study on limited-early response of DMO patients found 18% of patients satisfy the anatomic criteria, and 20% satisfy the visual criteria [11]. A study conducted by Singh et al. analysing the 36-month visual outcomes of DMO patients on ranibizumab found that those who gained <5 letters after 3 months showed a mean BCVA gain of +8.7 letters at 36 months, while those who gained 5–9 and ≥ 10 letters demonstrated letter gains of +14.3 and +19.5 at 36 months, respectively [12].

The anatomic outcomes of DMO are monitored using Optical Coherence Tomography (OCT) scans which measures CST [13]. OCT scans also assess the presence of intraretinal fluid (IRF) and subretinal fluid (SRF) which are poor visual prognostic indicators [14]. In DMO, anti-VEGF therapy has significantly reduced IRF, improving VA [15]. Notal OCT Analyzer (NOA) is a machine learning algorithm which quantifies IRF and SRF volumes. Kaakour and colleagues showed IRF and SRF were accurately quantified for DMO and retinal vein occlusion (RVO) patients [16]. Current studies utilize the NOA algorithm to quantify IRF and SRF levels of DMO patients [17].

The aim of this study is to characterize retinal fluid changes in DMO patients classified as limited-early or early responders to anti-VEGF treatment. The ML and image recognition algorithm will evaluate the residual fluid, BVA, and CST at 3 months, 6 months, and 12 months after initiating treatment and compare between responder groups. Furthermore, this study will help predict limited-early and early responders by evaluating baseline and 3-month outcome characteristics.

## Materials and Methods

This study has received approval from the Cleveland Clinic Foundation Institutional Review Board. Informed consent was not necessary due to the retrospective nature of the study. All procedures were performed in accordance with good clinical practice (International Conference on Harmonization of Technical Requirements of Pharmaceuticals for Human Use [ICH] E6), applicable FDA regulations, and the Health Insurance Portability and Accountability Act (HIPAA). A retrospective chart review was performed to identify early and limited-early responders to anti-VEGF injections of DMO patients.

This retrospective chart review analysed DMO patients at the Cole Eye Institute in the Cleveland Clinic between January 1st, 2012 and October 1st, 2019. All DMO patients in the study were ≥ 18 years and treatment naïve (*n* = 2503). 2233 patients were excluded because they received focal laser photocoagulation during the study period, had concurrent maculopathies, had a vitrectomy within 3 months of the study period, had an eye trauma within 6 months of the study period, or were pregnant during the study period. Patients were included if they had at least 12 months of follow-up and OCT scans taken at baseline, 3 months, 6 months, and 12 months after initial injection; 50 patients were excluded due to the lack of available OCT scans at these time points or presented with no fluid at baseline. If DMO was present bilaterally, only the first treated eye was included.

220 patients (220 eyes) were included in the study. After identifying the cohort, the Notal OCT Analyzer (NOA), a ML algorithm, quantified the SRF, IRF, and TRF volumes (nL). NOA distinguishes normal retinal features from elevated or distorted morphological features that occur due to fluid between the retinal layers [18]. Patients were then organized into limited-early responders (LERs) and early responders (ERs) based on the limited-early response criteria dictated in Piermaci et al.’s study: ≤ 10% CST reduction and/or <5 ETDRS letter gain at 3 months after initiating monthly anti-VEGF injections [11]. Piermaci’s criteria were utilized due to clinical relevance in routine practice and studies demonstrating varying long term outcomes between LER and ER in this classification [19]. The LER/ER group was further divided based on the limited-early responders who satisfied the anatomic criteria (aLER/aER) and the limited-early responders who satisfied the visual criteria (vLER/vER). Statistical analyses included the Levene test for homogeneity of variance of non-normal data, Kolmogorov-Smirnov test for normality, Welch’s t-test, Welch’s ANOVA, and one-way analysis of means.

## Results

### Characteristics and Summary

Of the 220 eyes (220 patients) included in the study, 162 were identified as LER while 58 were identified as ER based off Piermaci’s criteria mentioned above. In the subgroups of the limited-early and early responders, there were 113 LER who satisfied the anatomic criteria (aLER) and 107 who did not satisfy the anatomic criteria (aER), and 134 LER who satisfied the visual criteria (vLER) and 86 who did not satisfy the visual criteria (vER). 116 of the patients were male and 104 were female. Most patients identified as white and not Hispanic. Furthermore, 92.27% of patients were diagnosed with Type 2 diabetes mellitus, 73.63% had recorded insulin use, and 60% diagnosed with non-proliferative diabetic retinopathy (NPDR). Fisher’s exact tests found that there was no significant variance between all LER and ER groups.

Additional analysis was conducted to assess a possible correlation between LER status and anti-VEGF injections. The mean number of injections during the study period was 8.3; and there was no variance between the total number of injections and LER status (*p* > 0.1). Most patients (124) were treated with bevacizumab alone and several received bevacizumab with at least one other injection—aflibercept or ranibizumab (78). Analysis of bevacizumab injections revealed no significant variance by LER and vLER status (*p* > 0.1); however, there is a significant association between bevacizumab and aLER status indicating an impact on anatomical outcomes (*p* = 0.018). Aflibercept showed no significant variance compared to all LER definitions (*p* > 0.1). Additionally, analysis of ranibizumab and vLER status showed significant variance (*p* = 0.024). Further information on the demographics and breakdown between ER and all LER groups can be found in Table 1.

**Table 1:** Demographic Information of LER and ER.

		N	LER				ER
			All	Anatomic	Visual		
**TOTAL**		220	162 (73.64%)	113	134	58 (26.36%)	
**SEX**	Male	116	81 (69.83%)	54	64	35 (30.17%)	
	Female	104	81 (77.88%)	59	70	23 (22.12%)	
**RACE**	White	146	107 (73.29%)	68	90	39 (26.67%)	
	Black	55	43 (78.18%)	34	35	12 (21.82%)	
	Asian/Multiracial	9	4 (44.44%)	4	4	5 (55.55%)	
	Declined/Unavailable	9	8 (88.88%)	7	5	1 (11.11%)	
**ETHNICITY**	Hispanic	12	9 (75.00%0	7	7	3 (25.00%)	
	Not Hispanic	205	151 (73.66%)	105	125	54 (26.34%)	
	Unavailable	3	2 (66.66%)	1	2	1 (33.33%)	
**MEAN AGE**		61	61	61	61	60	
**LATERALITY**	OD	120	89 (74.34%)	58	76	31 (25.83%)	
	OS	100	73 (73.00%)	55	58	27 (27.00%)	
**DM TYPE**	Type 1	17	11 (64.7%)	9	9	6 (35.29%)	
	Type 2	203	151 (74.14%)	104	125	52 (25.83%)	
**INSULIN USE**	Yes	162	119 (73.46%)	80	98	43 (26.54%)	
	No	58	43 (74.14%)	33	36	15 (25.86%)	
**DR STAGE**	NPDR	132	100 (75.76%)	69	83	32 (24.24%)	
	PDR	88	63 (71.59%)	44	51	25 (28.41%)	
**MEAN # OF INJECTIONS**		8.3 ± 2.5	8.3 ± 2.6	8.3 ± 2.6	8.1 ± 2.5	8.2 ± 2.4	
**ANTI-VEGF INJECTION**	Bevacizumab alone	124	85	58	72	39	
	Aflibercept alone	18	10	3	9	8	
	Ranibizumab alone	2	2	2	2	0	
	Bevacizumab & Aflibercept	70	59	47	45	11	
	Bevacizumab & Ranibizumab	7	7	4	5	0	
	Aflibercept & Ranibizumab	0	0	0	0	0	
	Bevacizumab, Aflibercept, & Ranibizumab	1	1	1	1	0	

### Central subfield thickness and visual acuity outcomes

The baseline CST values were higher for all early response groups compared to the respective limited-early response groups, and significantly different for the aLER/aER and LER/ER groups (*p* < 0.01). The aLER and aER groups showed high significant difference in CST outcomes at 12-months with the aLER mean CST of 359.06 μm ± 134.93 μm and aER mean CST of 311.51 μm ± 88.49 μm (*p* = 0.0022). There were no significant differences of CST outcomes between vLER and vER groups and between LER and ER groups. The 12-month BVA outcomes showed a similar trend with the aLERs and aERs being significantly different. The aLER group had a 12-month BVA outcomes of 66.39 ± 13.46 ETDRS letter versus the aER group with 70.70 ± 12.46 ETDRS letters (*p* = 0.015). However, no significant differences in visual outcomes were found between LER and ER groups and the vLER and vER groups (*p* > 0.1). Mean BVA and CST at all time points are reported in Table 2 along with indications of values with significant variance from baseline.

**Table 2:** Mean VA and CST outcomes at each time point per LER/ER Group.

		Anatomic		Visual		Anatomic & Visual	
Month		aLER (*n* = 113)	aER (*n* = 107)	vLER (*n* = 134)	vER (*n* = 86)	LER (*n* = 162)	ER (*n* = 58)
0	VA (ETDRS)	65.06 ± 14.92	61.56 ± 14.35	69.02 ± 9.85	54.55 ± 16.65	66.08 ± 13.59	55.76 ± 15.20
	CST (μm)	384.00 ± 118.76	441.03 ± 134.79	403.32 ± 124.17	424.85 ± 137.59	398.09 ± 121.32	449.84 ± 145.10
3	BVA (ETDRS)	66.67 ± 13.90	69.15 ± 11.46*	65.87 ± 14.02	71.01 ± 9.92*	66.63 ± 13.54	71.35 ± 9.75*
	CST (μm)	401.78 ± 119.96	312.22 ± 78.05*	374.45 ± 119.23**	332.93 ± 91.86*	374.95 ± 115.72**	311.49 ± 80.61*
6	BVA (ETDRS)	67.068 ± 14.11	70.10 ± 11.62*	68.62 ± 12.68	68.42 ± 13.61*	68.08 ± 13.59	69.85 ± 11.26*
	CST (μm)	376.21 ± 128.86	325.19 ± 97.21*	342.71 ± 122.31*	342.94 ± 108.72*	358.47 ± 119.86*	331.64 ± 107.67*
12	BVA (ETDRS)	66.39 ± 13.46	70.70 ± 12.46*	67.80 ± 14.20	69.56 ± 11.28*	67.78 ± 13.72	70.47 ± 11.21*
	CST (μm)	359.06 ± 134.93	311.51 ± 88.49*	342.71 ± 130.40*	325.38 ± 91.78*	342.06 ± 124.49*	318.83 ± 91.28*

*significant variance from baseline (*p* < 0.001).

**marginal significant variance from baseline (*p* < 0.1).

When comparing CST values between time points, there was significant variance reported for all study groups. The vLER and LER varied with high significance between every time point (*p* < 0.001) and aLER varied with low significance between baseline and 3 months (*p* = 0.02), baseline and 12 months (*p* = 0.046), and no significance between baseline and 6 months (*p* = 1.00). The vLER group demonstrated variance between month 0 and 6 (*p* < 0.0001), and 0 and 12 months (*p* < 0.0001). The aLER group showed significant variance between months 3 and 12 (p < 0.0001). The LER group had significant variance between months 0 and 6 (*p* < 0.0001), and 0 and 12 (*p* < 0.0001). All early responder groups showed a high significance of variance between time points (*p* < 0.0001 for all). Each early responder group shows significantly high variance between baseline and all other time points (*p* < 0.000001).

Baseline VA was worse for all ER relative to LER groups, though only significantly difference for the vLER/vER and LER/ER groups (p < 0.0001). Visual outcomes were also compared between time points for all groups. The data found that BVA remained relatively constant throughout treatment for all limited-early response groups with very little variance between time points (*p* > 0.1). However, analysis indicated that there is variance between time points for all ER groups. The BVA was significantly different between baseline and all other time points, however no variance was noted between individual time points. The only significant variance was reported between baseline and month 3 for all groups (*p* < 0.001).

**Fig. 1:**
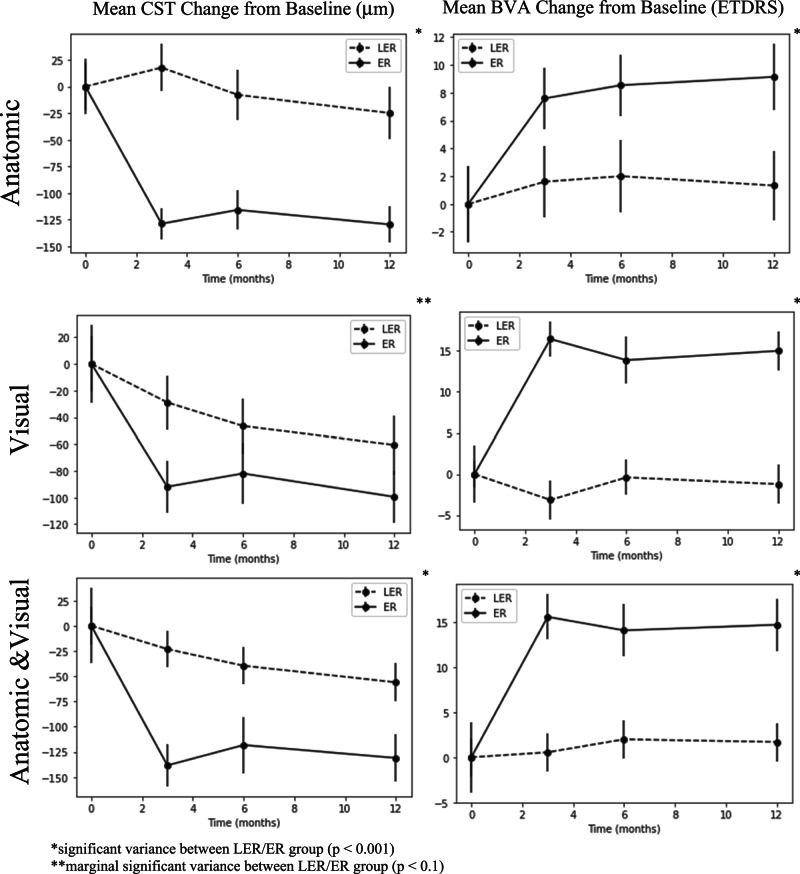
Mean BVA and CST Changes from Baseline per LER/ER Groups. *significant variance between LER/ER group (p < 0.001). **marginal significant variance between LER/ER group (*p* < 0.1).

The mean changes from baseline for CST and BVA were calculated and graphed for each LER/ER definition and shown in Fig. 1. Welch’s t-test analysis indicated that the mean CST changes from baseline were significantly varied by LER status for both the aLER/aER and LER/ER groups (*p* < 0.001) and showed a marginal significant variance for the vLER/vER group (*p* < 0.1). Analysis also found that mean BVA change from baseline significantly varied by LER status in all LER/ER groups (*p* < 0.001).

### Retinal fluid outcomes

Baseline retinal fluid volumes were higher for all ER groups relative to all limited early responders, though only IRF and TRF were significantly different in the ER/LER group (*p* < 0.01). The fluid outcomes at 12 months were not significantly affected by LER status by any definition for IRF, SRF, and TRF. However, 3-month SRF outcomes showed marginal variance by LER status for all definitions (*p* < 0.1); there was no variance in 3-month outcomes for IRF or TRF (*p* > 0.1). Mean retinal fluid volumes at each time point is reported in Table 3.

**Table 3:** Mean Retinal Fluid Outcomes at each time point per LER/ER Group.

		Anatomic		Visual		Anatomic & Visual	
Month		aLER (*n* = 113)	aER (*n* = 107)	vLER (*n* = 134)	vER (*n* = 86)	LER (*n* = 162)	ER (*n* = 58)
0	SRF (nL)	50.80 ± 223.87	63.91 ± 232.38	30.52 ± 109.91	50.80 ± 228.87	25.70 ± 100.48	74.03 ± 276.42
	IRF (nL)	1102.83 ± 1402.81	1216.72 ± 1391.45	770.29 ± 1069.24	1102.83 ± 1402.81	713.84 ± 1008.39	1421.04 ± 1567.45
	Total RF (nL)	1153.63 ± 1504.26	1280.64 ± 1480.06	800.81 ± 1104.69	1153.63 ± 1504.26	739.55 ± 1040.89	1495.07 ± 1687.67
3	SRF (nL)	23.78 ± 87.83	5.85 ± 30.94**	21.50 ± 84.06	5.03 ± 18.49	19.24 ± 77.35	3.37 ± 12.56
	IRF (nL)	677.46 ± 817.13	508.28 ± 704.56*	638.97 ± 822.19	526.95 ± 672.23*	616.70 ± 789.23	535.06 ± 705.95*
	Total RF (nL)	701.23 ± 878.13	514.14 ± 719.84*	660.47 ± 880.73	531.97 ± 679.05*	635.95 ± 842.24	538.43 ± 705.95*
6	SRF (nL)	17.97 ± 67.34	13.32 ± 96.32	16.26 ± 63.43	14.86 ± 106.20	14.15 ± 58.13	20.08 ± 128.99
	IRF (nL)	596.07 ± 847.85	528.96 ± 797.15*	550.85 ± 829.77	559.42 ± 814.75*	527.06 ± 793.63	630.00 ± 899.74*
	Total RF (nL)	596.07 ± 896.76	542.28 ± 797.15*	567.11 ± 873.3276	574.28 ± 857.75*	541.21 ± 932.89	650.08 ± 953.18*
12	SRF (nL)	6.16 ± 24.29	2.71 ± 14.04**	5.74 ± 22.53**	2.51 ± 15.18	4.79 ± 20.59**	3.61 ± 18.42
	IRF (nL)	548.76 ± 1274.17	484.4 ± 712.69*	570.13 ± 1237.63	435.40 ± 606.15*	520.10 ± 1143.82	510.10 ± 665.67*
	Total RF (nL)	554.92 ± 1287.95	487.12 ± 7122.69*	575.87 ± 1249.94	437.92 ± 610.11*	524.89 ± 115.02	513.71 ± 970.64*

*significant variance from baseline (*p* < 0.001)

**marginal significant variance from baseline (*p* < 0.1)

The analysis of TRF variance across time points revealed no significant variance for all limited early response groups (*p* > 0.1), however there is significant variance for all early responder groups between baseline and all other time points (p < 0.0001), though not between time points (*p* > 0.1). The IRF analysis showed a similar trend as TRF with no significant variance across all limited early response groups (p > 0.1) and with high significance in all early responder groups between baseline and all other time points (*p* < 0.0001). However, SRF showed marginal variance between baseline and 12 months for the vLER and LER groups, but no significant variance between any time point for the aLER group. Between all early responder groups, SRF only varied for the aER between baseline and 3 months with marginal significance (*p* < 0.1).

## Discussion

Results indicated that 12-month outcomes for retinal fluid, BVA, and CST were not significantly different between limited-early response and early response groups. However, all early response groups demonstrated significant improvement in their VA, CST, and retinal fluid volumes by 12 months relative to changes in respective limited-early response groups suggesting better anatomic and visual outcomes for the early response groups. Furthermore, the ER group showed the largest improvement in CST and BVA between baseline and 3 months after initiating injections indicating that the change in BVA and CST by 3 months is indicative of 12-month outcomes.

By month 12, the aLER and aER groups had significantly different outcomes relative to the other limited-early and early response groups indicating that those with limited CST reduction at 3 months after initial monthly injection had worse BVA and CST outcomes 12 months after initiating treatment. This finding is consistent with current DMO studies demonstrating a moderate correlation between worse CST and worse BVA [20, 21]. Time variance analysis and Fig. 1 demonstrates the greatest BVA change occurred within the first three months of initiating anti-VEGF injections suggesting limited BVA improvement after 3 months for all limited-early and early responders likely due to reacting to initial anti-VEGF treatment noted in Piermaci et al. study [11]. Furthermore, all early response groups had significantly higher mean ETDRS letter gain and mean CST improvement indicating that all early responders have better VA and CST outcomes 12-months after initiating treatment. Dugel et al.’s study—which categorized limited-early responders as those with ≤ 20% central retinal thickness (CRT) reduction at 12 weeks after initial anti-VEGF injection—similarly found that early responders had significantly better BVA and CRT improvement at 52 and 156 weeks [13]. The data can be explained by the floor and roof effect due to better anatomical and visual baselines in limited-early response groups in both studies. Since the limited-early response groups had better baseline values than their early response counterparts, they had limited room for improvement compared to the early responders. Further study into the effect of baseline CST and VA on LER/ER outcomes could provide more insight into their impact on long-term outcomes.

Moreover, CST and BVA change appears more gradual within the limited-early response groups relative to the rapid change seen in early response groups (Fig. 1). Time variance analysis of CST and BVA in limited-early response groups revealed significant improvement between every time point not just a very high improvement between baseline and others as noted in early response groups. This result indicates that the limited-early responders may continue to improve CST and BVA after the 12-months or remain constant. Piermaci et al.’s study found that limited-early responders continued to improve CST and BVA outcomes at about 100 weeks after initiating anti-VEGF treatment [11]. However, current long-term CST and BVA outcomes between limited-early and early responders have varied between multiple studies. Further study into CST and BVA changes past 12 months of initiating anti-VEGF in limited-early responders may clarify if this group displays a gradual change over time or a limited improvement overall.

Retinal fluid outcome findings in this study varied by fluid location. While 12-month outcomes for all retinal fluid outcomes did not vary LER status in any group, SRF 3-month outcomes marginally varied by LER status for all limited-early and early response definitions. By 3 months, all early responder groups had reduced SRF volume to around 5 nL while limited-early responders reduced to around 20 nL as shown in Table 3. This finding correlates with early responder groups showing the greatest CST reduction between baseline and 3 months. Since CST is used to assess retinal fluid and monitor macular oedema, retinal fluid reductions may mirror the CST reductions. Time variance analysis revealed vLER and LER groups showed SRF outcomes significantly varied between baseline and 12 months. The aLER demonstrated an improvement in the mean SRF volume between baseline and 12 months though not significant. Since the mean SRF volumes decrease over time in these two groups, SRF improvement may indicate gradual improvement mirroring CST outcomes and its gradual improvement noted in the respective limited-early response groups. However, the VIVID/VISTA Phase III studies found that SRF volume had decreased to nearly 0 within the first 12 weeks of treatment which is inconsistent with our findings [21]. Majority of studies have evaluated the impact of baseline SRF on DMO treatment outcomes, though not many have explored how SRF outcomes change throughout anti-VEGF treatment in DMO patients and require further study.

IRF and TRF outcomes showed significant improvement for all early response groups at month 3 but had limited improvement after while all limited-early responder groups showed no significant improvement after baseline indicating limited to no improvement. This result mimics CST and BVA outcomes in early responders showing most improvement between baseline and 3 months. This trend is consistent with studies demonstrating higher levels of IRF, SRF, and TRF correlate with worse BCVA outcomes at 12 months after initiation of anti-VEGF injections in DMO patients [17]. The relationship between retinal fluid, CST, and BVA results can be explained by the negative impact of sustained retinal fluid on the ellipsoid zone (EZ), disruption of which is directly correlated to the severity of DR [22]. Additionally, increased CST has been shown to increase the odds of EZ disruption [23]. 12-month outcomes for all retinal fluid demonstrated no affect by limited-early response status which mirrors the 12-month outcomes seen with BVA and CST. Since baseline retinal fluid values were worse for all early response groups with significant improvement compared to their respective limited-early response groups, the trend can also be attributed to the floor/ceiling effect as mentioned above, mirroring CST and BVA outcomes.

The study found no variance between the demographics and all LER definitions; however, some variance was found with the anti-VEGF injection type. Bevacizumab showed a significant difference between the aLER and aER groups indicating that bevacizumab may be associated with gradual anatomic changes in the aLER group as noted above. The Protocol T Extension study confirms this finding by demonstrating bevacizumab injections were associated with worse anatomical outcomes as compared to aflibercept and ranibizumab [24]. Additionally, ranibizumab showed significant difference between the vLER and vER groups indicating its possible impact on VA outcomes. However, no full conclusions on ranibizumab’s VA outcomes can be measured because only 2 limited-early response patients received only ranibizumab injections and no early response patients received any ranibizumab with or without the other injection types. Additionally, Protocol T did not discover worse VA outcomes with ranibizumab compared to the other injections which is inconsistent with our data [24]. Further study of anatomic and visual outcomes of DMO patients with an analysis of anti-VEGF type would clarify current findings on impact of injections on LER status.

This study contains some limitations that should be noted. Of the 220 patients collected in the retrospective analysis, most participants identified as White and Not Hispanic. With significantly smaller groups in other races and ethnicities, no conclusions could be drawn on limited-early/early response status relative to these baseline characteristics. Furthermore, no demographic baseline characteristics were statistically significant between groups, which allowed us to assess data while controlling for factors but may not be representative of all DMO patients. Future studies should investigate the possible effect of baseline characteristics and demographics on limited-early response status. Furthermore, while our analysis revealed the impact of the floor and ceiling effects on all limited early response groups due to better VA and CST at baseline, further study into LER patients with worse baseline CST and VA values may indicate demographic characteristics that affect LER status. No data following the 12-month outcome was available for analysis, therefore, analysis of long-term outcomes past 1 year cannot be concluded. Additionally, the data from OCTs are from the NOA and anatomic characteristics such as disorganization of retinal inner layers were not used and thus no analysis was done. Another subject of note is the unequal sizes of the LER and ER populations. This study found 162 patients (73.6%) classified as limited early responders, whereas most studies reported 40–50% LER, such as Piermaci et al. with 43.5%, 14.8%, and 11.5% of each group satisfied the limited early responder criteria [11]. The variation may be accounted for by the differing study inclusion and exclusion criteria such as Piermaci et al. only including patients with CST values ≥ 300 μm at baseline [11]. As a result, the mean baseline CST is higher for both the limited-early and early responders in Piermaci et al.’s study, and therefore, the smaller percentage of limited-early responders is likely due to the floor effect. Our analysis has accounted for the significant variance in population size, but the large difference in two groups should be noted.

Altogether, all ER groups showed significant improvement in CST, BVA, retinal fluids between baseline and 3 months and remained relatively constant to 12 months while all LER groups showed limited improvement between baseline and 3 months and gradually improved by 12 months. These results indicate that limited-early/early response status determined at 3 months after initiating anti-VEGF treatment can predict long-term outcomes. Baseline CST, BVA, and retinal fluid in LER groups may explain the limited improvement relative to ER groups due to the floor/ceiling effect. Furthermore, EZ disruption is negatively affected by CST and retinal fluids which helps explain the BVA outcomes. These conclusions will help physicians understand and utilize trends in retinal fluid, VA, and CST in limited-early and early responders for DMO treatment. Further studies exploring the effect of SRF throughout DMO treatment period and analysis of long-term outcomes beyond 12-months are necessary to draw further conclusions to the findings.

### What was known before

- Patients with diabetic macular oedema have various treatment response to anti-VEGF injections. Current studies demonstrate that the treatment response can be characterized as limited-early or early response at 3 months in regard to CST and VA improvement at this time point. Some studies have also explored the impact of the limited-early and early responders on long-term treatment outcomes, but requires additional studies.

### What this study adds

- This study investigates the impact of intraretinal, subretinal, and total retinal fluid on limited-early response status to determine the role of fluid in DMO. Additionally, the study divides the groups into anatomical (CST) and visual (VA) limited-early responders to determine additional factors that may affect their treatment outcome and the effect of their status on long-term outcomes.

## Data Availability

The raw datasets generated during the study are available from the corresponding author on reasonable request. All data analysed during the study are included in this published article and its supplementary information files.
